# Measuring outcomes of a successful mentoring dyad: A case study to optimize resources

**DOI:** 10.1097/01.NUMA.0000754096.40272.05

**Published:** 2021-06-28

**Authors:** Judith T. Caruso, Kathleen Perez

**Affiliations:** **Judith T. Caruso** is the president and CEO of Caruso Consulting in Morristown, N.J., and **Kathleen Perez** is the administrative director of medical-surgical nursing at Hunterdon Medical Center in Flemington, N.J.

## Abstract

The Organization of Nurse Leaders of New Jersey has had a formal statewide nurse leader mentoring program since 2014 to develop leadership talent. Learn how a mentoring dyad helped them improve competencies in optimizing resource management.

**Figure:**
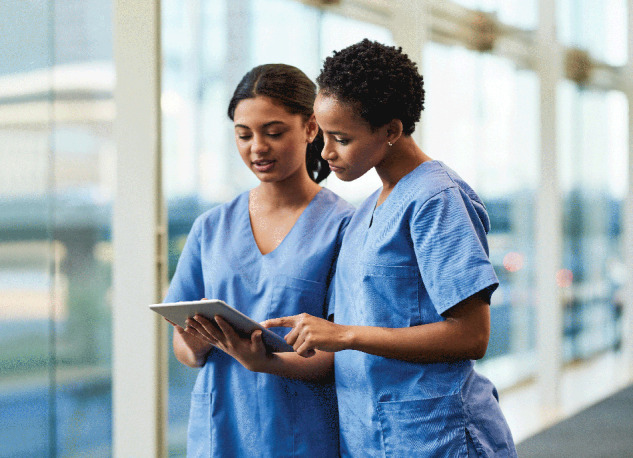
No caption available.

The Organization of Nurse Leaders of New Jersey (ONL NJ) has had a formal statewide nurse leader mentoring program since 2014 to develop leadership talent. Mentorship is incorporated into the ONL NJ strategic plan and continues to be active at ONL NJ.^1^,^2^ The biannual ONL NJ Mentoring Program is designed as a 1-year mentoring experience for leaders at all levels and in various capacities, serving over 100 dyads to date. See Table 1 for a summary of the research results that guided the updated focus of the mentoring education sessions.^2^ A structured educational program offered biannually along with the ONL NJ Mentorship Tool Kit has provided many tools for success for each dyad to understand the phases of the mentoring relationship.^2^-^4^

**Table 1:** ONL NJ Mentoring Program education workshop: Mentorship committee activities^2^

Session	Activities	Purpose
Preeducation	Collect résumé/survey of mentors and mentees Organize seating arrangements of education session	Identify common interests/specialties/expertise Consider geographic location, mentoring goals, current positions
Education	Curriculum includes understanding of mentoring relationships, being reflective and aware, and an introduction to the Mentorship Tool Kit and its components Incorporation of small group projects Table assignments strategically made based on potential pairings Participants list potentials for pairing	Provide meaningful instruction on what mentoring is, its goals and objectives, and how to make it beneficial for both participants Facilitate networking and relationship building among potential pairings Support natural personal connections
Posteducation	Committee review of pairing requests Notification of pairing with distribution of tool kit Quarterly follow-up by the committee liaison to facilitate successful pairings	Provide as much support and guidance as possible

However, not all mentoring dyads work out.^2^,^4^ This article discusses the successful outcomes of a dyad from the 2018-2019 ONL NJ Mentoring Program cohort who focused on improved competencies in optimizing resource management.

## Establishing the mentoring relationship

The mentee (author Perez) is an administrative director of medical-surgical nursing at a 178-bed general medical and surgical teaching hospital in New Jersey. The mentor (author Caruso) is a healthcare consultant focused on linking financial and clinical data for improved outcomes.

In the mentee's current position as administrative director of medical*-*surgical nursing, she wanted to enhance her capabilities in optimizing resources and controlling salary expenses. She sought a mentorship relationship that would help increase her financial understanding and competencies in the fiscal management of human resources, a key managerial competency as described by the American Organization for Nursing Leadership (AONL) in the domain of The Science: Managing the Business.^4^,^5^

As members of ONL NJ, both authors as mentee and mentor attended mentoring educational seminars offered through ONL NJ. The mentee reached out to the mentorship committee using a specific form to request a mentor “match” based on her goals. The mentor had done mentoring in the past and was looking to mentor another mentee as part of cohort 6 of the ONL NJ Mentoring Program. ONL NJ then sent both authors copies of their respective résumés for review and included the mentee's goals. For the past 25 years, the mentor has had an extensive background in nurse staffing and optimizing management of resources, focusing on linking financial and clinical data for improved outcomes. As Goodyear and Goodyear reported, effective mentoring relationships depend on the skills of the mentee and mentor.^6^,^7^ In addition, qualitative research reported by Vitale indicated that mentoring success is built on common interests, evaluating/reevaluating goals driven by the mentee, and effective communication.^2^

In June 2018, the mentee was informed that she'd been selected to participate in cohort 6 of the ONL NJ Mentoring Program and was matched with her mentor. In August 2018, the mentee and mentor scheduled a first meeting to start cultivating the relationship, review goals, and develop a plan of action for future meetings. The first 3 months of meetings in 2018 were focused on the mentee's objective to better understand internal budget and financial reports regarding human resources.

The key stated goals were to:

- manage the operating budget daily, which included all full-time equivalent (FTE) personnel and all expenses for day in/day out operation of her three medical-surgical units
- ensure that actual productivity of nursing hours per patient day matched budgeted productivity of nursing hours per patient day and provide further analysis and understanding of variances to budget.

Additional objectives added during the first 3 months included to:

- reduce nonholiday overtime (OT) hours and expenses of RN staff
- use newly developed competencies and expertise to teach and mentor her own three directors with their clinical coordinators to better manage their staff resources.

Scheduling regular meetings and planned communication is essential to a successful mentoring relationship.^2^,^6^,^7^ The mentor and mentee met once a month for 1 hour in person or by phone for 1 year, scheduling the next meeting at the end of each meeting. The mentee prepared a mentoring meeting agenda. Each meeting started with a “How are you doing?” approach. This created an opportunity to openly discuss what had happened in the previous month and what challenges or barriers the mentee was experiencing. In positive mentor-mentee relationships, it has been recognized that mentees need to determine the goals and manage the process that will be most successful for them.^3^

## Cultivating the relationship

The mentee had a unique opportunity to develop the business knowledge and skills needed to be successful in her career. The mentor helped the mentee identify areas in the nursing budget process that she needed to improve. In working together, the mentor helped the mentee realize that budgets and cost analytics don't have to be as stressful as she thought. The mentor always provided a positive learning atmosphere while providing the mentee with useful tools, valuable knowledge, and new expertise to manage resources.

The dyad first reviewed the staffing budgets of the mentee's three medical-surgical units. As the mentor explained, you can't understand a variance report to budget if you don't fully understand the personnel budget.^8^ The mentor and mentee discussed the agenda, which included reviewing reports generated from the hospital's financial and budget system, focusing on budget versus actual numbers for staffing and budget assumptions.

The mentor provided additional tools with an electronic spreadsheet that she developed showing how a master staffing plan can be used for budgeting purposes to fully understand how productivity of nursing care hours is calculated, how staffing patterns dictate the FTEs needed, and how to calculate specific RN positions needed based on weekend needs and total FTEs. This increased understanding gave the mentee the confidence to seek additional information from the finance department on certain data to better understand these data and use them more fully with her manager direct reports. Often, new nurse leaders “don't know what they don't know” when they try to deal with budgets and new finance and gain understanding as they try to “connect the dots.”^8^

Next, they reviewed the various reports on staff productivity to increase the mentee's understanding of her own organization's financial reporting tools of variance analysis. She expressed concern with the high nonholiday RN overtime OT usage, not yet understanding what caused the high OT and how to implement procedures to decrease OT expenses.

At the November 2018 dyad meeting, the mentee included one of her clinical directors, who was having excessive OT usage, to do a “deep dive” into the clinical director's unit budget and understand the root cause behind the high OT usage. At the time, this clinical director hadn't fully understood the staffing plans, the budget, or how best to control OT usage. Further analysis revealed that there was a pattern of “casual” OT (time adding to OT from early punch-ins and late punch-outs). There was also a concern regarding the use of full-time RNs for extra shifts and low use of per diem nurses and part-timers, increasing OT hours.

At about 6 months into the mentoring experience, the mentee wanted to put action plans into place with her own clinical directors and their clinical coordinators to begin the process of reducing OT costs for their new fiscal year of 2019. However, before an action plan could be fully designed and implemented, the clinical director, clinical coordinators, and the RN staff formed a task force to work on a performance improvement plan. The leaders have a strong shared governance model in place and realized that the success of this endeavor needed to start with the challenges as viewed by the staff.

The task force agreed on the actions needed and began implementation in several areas:

- Reduce “casual” OT (hours worked either before or after a scheduled shift that weren't officially OT until the employee worked 40 hours a week). For example, if a full-time employee punched in early or punched out late for a total of an extra 4 hours a week beyond their scheduled three 12-hour shifts (36 hours) a week, their total paid hours would be 40 hours, 36 of the hours budgeted, but another 4 hours not budgeted. These 4 hours could account for 4*/*36 (11%) extra paid regular productive hours above what was budgeted. The leaders and RN staff were surprised by how costly these extra hours could be before the hours over 40 were even counted as OT hours. They couldn't believe that this could be an additional 11% above budget when it occurred. They agreed that excessive hours needed to be addressed to control costs, as well as nurse fatigue when working beyond a 12-hour shift.^9^
- Reduce nonholiday OT hours greater than 40 hours a week by scheduling extra shifts in a less costly manner (schedule per diem and part-time staff before use of OT hours from full-time RN staff).
- Begin end-of-shift huddles about 5 p.m. each day with clinical coordinators on day shifts and RN staff to determine who might be feeling overwhelmed and anticipating not being able to complete all work on time. The clinical coordinators and RN staff then focused proactively on how they could assist their RN colleagues to complete the work as a team. These huddles identified workflow issues, learning needs of staff, and the impact of clinical process issues with other departments that needed to be addressed. Twelve-hour shifts are exhausting and working longer than 12 hours doesn't promote patient and employee safety.^9^

## Implementation of action plans

The mentee's institution is committed to actively creating and promoting a healthy work environment that fosters and supports excellence in patient care. It's one of 37 New Jersey hospitals participating in an ONL NJ initiative for nursing workplace environment and staffing councils to bring about positive change, with clinical nurses taking the lead. This work is under the conceptual framework of the American Association of Critical-Care Nurses' *Standards for Establishing and Sustaining Healthy Work Environments*, which has six interdependent standards: skilled communication, true collaboration, effective decision-making, meaningful recognition, authentic leadership, and appropriate staffing.^10^ The nurse leaders and staff members' desire to work on this OT cost reduction initiative drew from the strong principles in improving their work environment and promoting greater teamwork.

During the months of this work together, the leaders and staff continued to monitor their unit quality dashboards and staff engagement. They didn't experience declines in quality measures and improved in some staff satisfaction data as noted from 2 years of National Database of Nursing Quality Indicators^®^ (NDNQI^®^) staff satisfaction scores. (See Table 2.)

**Table 2:** NDNQI job enjoyment scale results∗

Unit	Hospital NDNQI job enjoyment score: 2017∗∗	Hospital NDNQI job enjoyment score: 2019∗∗
#1: Medical ADC = 28	3.66 (slightly below the national NDNQI mean of 3.95)	3.77 (improved but below the national NDNQI mean of 3.93)
#2: Surgical ADC = 22	3.96 (slightly below the national NDNQI mean of 3.97)	4.42 (improved, above the national NDNQI mean of 4.01)
#3: Progressive care ADC = 21	3.89 (at the national NDNQI mean of 3.89)	3.70 (decline, below the national NDNQI mean of 3.93)

∗ Measure of the degree to which RNs like their work based on seven questions (2-year comparison)

∗∗ Scoring: 1 to 6 (strongly disagree to strongly agree)

**Figure 1:**
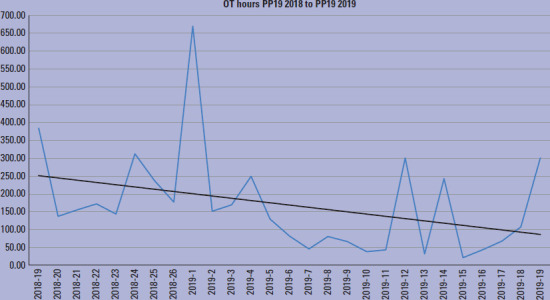
Unit #1 (medical): Trending down of RN OT by 60%

**Figure 2:**
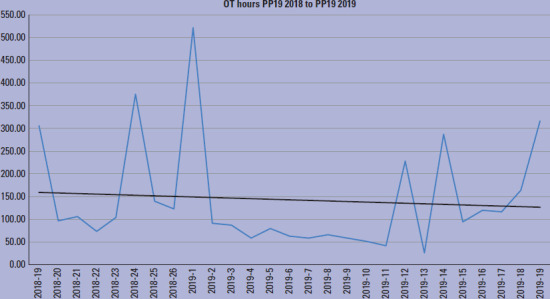
Unit #2 (surgical): Trending down of RN OT by 13%

**Figure 3:**
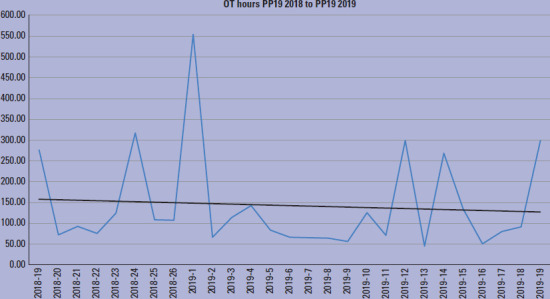
Unit #3 (progressive care): Trending down of RN OT by 12%

The mentee shared personnel budget variance reports with her mentor and had conference calls to determine progress made in implementing plans to control costs from their interventions. The mentee shared how the actions by her clinical directors and their teams decreased “casual” OT, reduced unnecessary OT, and continued to trend downward. (See Figures 1, 2, and 3.)

## Successful outcomes: Reduced OT usage and more teamwork

As reported in the literature, it's critical for mentees to take responsibility for their own development and be self-reflective of their ability to learn and grow.^2^,^3^,^6^,^7^ This dyad cultivated a relationship through time, energy, and resources to develop, teach, and mentor as reported by other successful dyads.^11^ In this case study, both the mentee and mentor had a positive experience and truly found joy in the work.

When it comes to fiscal management, nurses tend to feel intimidated.^8^ The mentee stated that she grew in fiscal budgeting knowledge and optimizing use of resources beyond her original goals of what she thought she could achieve. In addition, she was able to use her improved knowledge and skills to better mentor her own clinical directors, develop a stronger relationship with the finance department, and gain use of more meaningful reports for the managers to better optimize their resources.
